# Neurofunctional Evaluation of Young Male Offspring of Rat Dams with Diabetes Induced by Streptozotocin

**DOI:** 10.5402/2011/480656

**Published:** 2011-09-22

**Authors:** Carla Delascio Lopes, Rita Sinigaglia-Coimbra, Jacqueline Mazzola, Luiz Camano, Rosiane Mattar

**Affiliations:** ^1^Departamento de Obstetrícia, Rua Napoleão de Barros 875, Universidade Federal de São Paulo, 04024-002 São Paulo, SP, Brazil; ^2^Laboratório de Neuropatologia e Neuroproteção, Rua Pedro de Toledo 781, 7 andar, Universidade Federal de São Paulo, 04039-032 São Paulo, SP, Brazil

## Abstract

Diabetes mellitus (DM) is a complex disease, being one of the most prevalent diseases worldwide. As a consequence, pregnancy-associated diabetes is increasingly common. Given the numerous studies about the influence of diabetes on offspring of diabetic rat dams, the neurological outcome is of outmost importance. This paper aimed at evaluating the neurofunctional performance of young male offspring of rat dams with diabetes induced by streptozotocin. Diabetes was induced in Wistar female rats by streptozotocin administration, while control groups received vehicle injection. At two-month survival period, male offspring from each group were randomized to the water maze Morris test, in order to assess their neurofunctional status. There was no significant difference between the groups as assessed by the Morris water maze test for spatial reference task. Our results point to the need of further investigation on the offspring neurofunctional performance.

## 1. Introduction

Diabetes is the most common metabolic disturbance during the pregnancy cycle [1], being an important cause of maternal fetal morbidity [2]. The incidence is 7% at the gestation [3], being the metabolic complication highly associated to maternal and fetal risks [4]. Concerning the prevalence, it is very variable, with index varying from 1 to 14% of all gestations. It is influenced by the ethnical and racial population distribution and by the chosen method for the tracking and diagnosis [5]. The gradual increase of this occurrence, which can be explained by the increase of mother's age and weight average [6] has been observed. 

The central nervous system (CNS) is particularly vulnerable to the intrauterine hyperglycemia, being the malformation risk approximately 15.5 times higher in diabetics mothers [7]. CNS development is not restricted to organogenesis, as the cerebral cortex still suffers changes in the postnatal period [8]. Despite the high prevalence of diabetes, little is known about its effects on the CNS during fetal development, and what are the cognitive sequelae. 

In humans, children from diabetic mothers may exhibit abnormalities, which include motor difficulties, attention deficit, learning defects, and also the risk of developing schizophrenia during adolescence [9–12]. 

Plagemann and colleagues [13] reported that offspring from diabetic dams have high levels of dopamine and norepinephrine in the hypothalamus, increased dopamine, norepinephrine and serotonin at the caudate nucleus, as well as brain weight reduction.

However, little information is available on the neurofunctional performance of offsprings from diabetic rat dams. This work aimed at evaluating the neurofunctional behavior (cognitive and motor) of 60-day-old male offspring from streptozotocin-induced diabetes rats as assessed by the water maze Morris test.

## 2. Materials and Methods

This work is an experimental, analytical, prospective, and controlled study. Young adult female Wistar rats were obtained from the Center for Development of Experimental Models (CEDEME, UNIFESP), weighing 200–250 g, and maintained in pressurized cages on a 12 hr light/12 hr dark cycle (lights on from 7:00 AM) at room temperature between 22.0 and 24.0°C with free access to food and water. The offspring stayed 60 days at the same conditions until the end of the neurofunctional evaluation. 

According to Calderon [14], the following experimental sequence for the study of diabetes in pregnant Wistar rats was adopted, comprising four periods: (a) adaptation, (b) diabetogenic, (c) mating, and (d) pregnancy. Briefly, these four periods comprise (a) adaptation to the laboratory condition for 7 days and (b) hyperglycemia induction obtained by streptozotocin i.p. injection at the dose of 50 mg·Kg^−1^, diluted in 0.3 mL of 0.1 M citrate buffer [15–17]. During the first 72 hours, a 5% glucose solution was given as a water substitute to avoid hypoglycemia caused by hyperinsulinemia. From the third day of the experiment, the rats had free access to water and diet ad libitum. At the same period, glycemia was determined to assure the presence of the hyperglycemic state. The glycemia was assessed by the puncture of tail vein with the aid of a glycemia monitor (Glucotrend 2, Roche). Only rats with glycemic values equal or higher than 250 mg/dL were considered as diabetics [17, 18]; (c) after confirmation of elevated glycemic index, the rats were transferred to cages for mating for 15 days (one male to five females). Daily vaginal cytology was performed to confirm fecundation; (d) the pregnant rats confirmed as diabetic at first day of pregnancy were separated and kept in individual cages until the birth of the offspring (20–22 days) and suckling period (21 days). Body weight gain control was daily assessed. 

The pregnant rats were separated into two groups: (a) Streptozotocin Group (STZ): STZ-induced diabetes (*N* = 10); and (b) Control Group (CTRL): rats receiving vehicle solution (citrate buffer 0.1 M, pH 4.5). Glycemic indexes were verified at morning without fasting at the mating day to confirm the hyper- or normoglycemia, as well at the first day postpartum. Only 50% of diabetic rats became pregnant (five out of ten), and after the birth, one of the dams did not sustained hyperglycemic levels. For this reason, it was removed from the study. The offspring was separated in cages according to the sex. At two months old (approximately 200 grams of weight), the male offspring from each group were randomly selected (one or two male rat from each dam, depending on the number of male offspring obtained from each dam). 

The pregnancy success index was higher at the CTR group, reaching 70% (7 out of 10). Again, one or two male rats from each normoglycemic dam were randomly selected. The selected male offspring of each group were submitted to neurofunctional evaluation at 60 days old: (a) offspring of diabetic rats (C-STZ), *N* = 7; and (b) offspring of nondiabetic rats (C–CTRL), *N* = 14. 

## 3. Neurofunctional Evaluation

At 60 days of age, motricity and cognition of the offspring were evaluated, by the water maze Morris test for spatial reference task [19]. Testing occurred in a 2-meter diameter black pool centered within a rectangular room. An overhead camera was connected to a video monitor and a computer running the software (Ethovision 2.3, Noldus Information Technology, Netherlands) used to track the rat swimming path, and to calculate the path length and the time (latency) spent to reach an invisible (black) platform placed 1 cm under the water surface. Each animal was tested four times a day for seven consecutive days. For scoring purposes, the pool was divided in four quadrants. The platform was placed in the middle of one specific quadrant, for all testing. The animals were released into the pool from each of 4 starting locations daily, in a pattern that was randomly determined prior to testing. For every trial, the animal was placed in the pool facing the wall. Animals were allowed 120 s to find the platform. If they were unable to find the platform in that time, they were guided to it by hand. They were allowed to remain upon the platform for 30 s and were then removed. Visual cues were available within the testing room. A minimum of 5 min elapsed between trials, during which time the animal was placed under a heat lamp, on an elevated platform in the testing room. All testing was started by 7:00 AM. Repeated measures analysis of variance for latency was done, followed by Dun-Sidak's test. Mean latency ± SEM of each day (session) was obtained for the purpose of data representation.

## 4. Results and Discussion

This study used dams with streptozotocin-induced diabetes as biological model to evaluate the neurofunctional status of male offspring at 60 days old. No signs of motor or cognitive impairment could be detected in our experimental design. However, these results are not conclusive, since only offspring from the 50% of successful dams were tested in our study. 

The neurocognitive development of children from mothers with compensated pregestational diabetes is similar to that observed in children from normoglycemic mothers [20]. Therefore, the glycemia maintenance under normal values is associated to the decrease of adverse perinatal results, like fetal abnormalities, macrosomia, fetal death, and neonatal complications [13]. However, the inadequate control of the disease can cause cognitive and motor prejudice to the offspring. Petersen et al. [21] described that diabetic women, mainly those with the type 1, have higher risk to present intrauterine growth restriction and malformations, contributing to neuropsychomotor development delay. Contradictorily, pregestational diabetic or compensated gestational diabetic mothers can generate children with psychomotor disorders [20].

The main neurological changes, observed in children born to diabetic mothers, are hyperactivity, attention deficit disorders and delayed motor development [22]. The neurological development of infants to diabetic mothers has been studied for nearly 40 years. Churchill et al. [23] were the first to describe the lower scores for intelligence quotient (IQ) in children to diabetic mothers exhibiting ketonuria, against the normal IQ scores for children to controlled diabetic mothers. Stehbens et al. [24] studied children to diabetic mothers at 1, 3, and 5 years old. The authors reported higher mortality and lower cognitive scores in children born small for gestational age when compared to control group. This finding was also confirmed some years later by Petersen et al. [21]. In contrast, Cummins and Norrish [25] found no differences in relation to cognitive scores in children of diabetic mothers between 4 and 13 years old. Persson et al. [26] also confirmed these findings in children under 5 years old. 

The aspects related to the neuropsychomotor and cognitive developments of the offspring to diabetic dams, specifically in young male rats, were investigated. We decided to restrict the neurofunctional evaluation to this group due the hormonal influence, since some experimental evidence showed that the physiology and anatomy of the nervous system suffer fluctuations according to the estral cycle of the rats [27–30].

**Figure 1:**
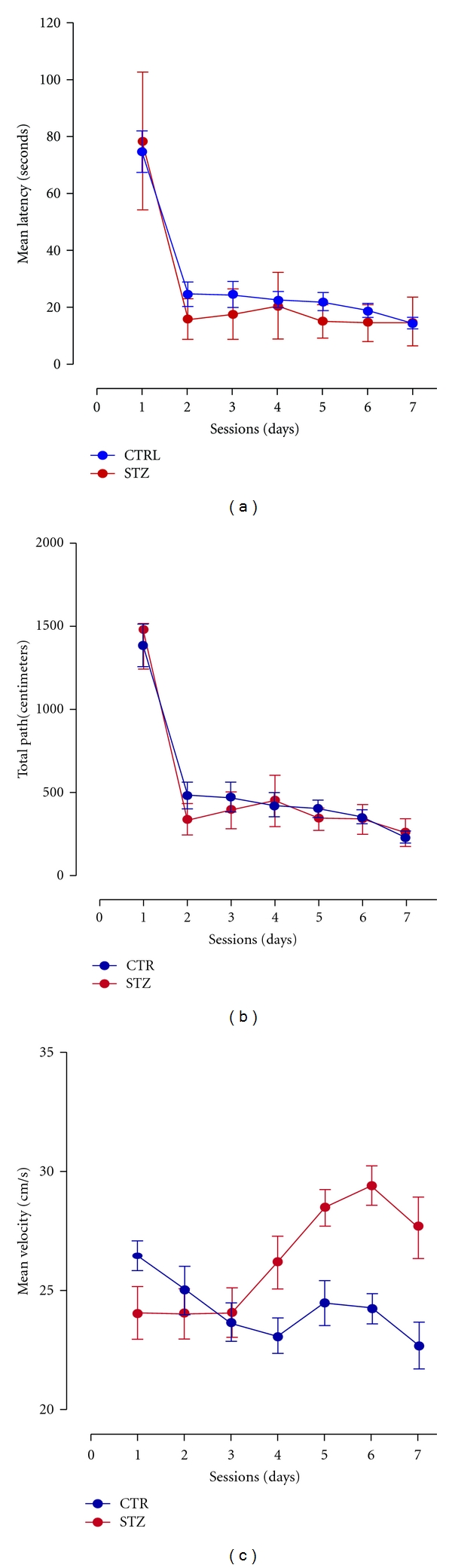
Performance of young male offspring to diabetic dams and normoglycemic dams in the Morris water maze test for spatial reference memory at 60-day survival. Mean escape latency (a), total path (b), and mean velocity (c), series of 4 daily trials during 7 consecutive days. No statistical significance was found between groups (ANOVA).

The water maze Morris test is considered well discriminative and valued in the specialized literature, allowing the researchers to dissociate memory impairment from deficits sensory, motor, motivational, and retrieval processes [31, 32]. In this experiment, the neurofunctional evaluation (cognitive and motor) using this test did not show a significant difference between the studied groups (Figure 1), in the three evaluated parameters (latency, path, and speed). The absence of neurocognitive repercussions on the male offspring to diabetic mothers is certainly puzzling, in accordance to the literature showing controversial results with human studies. 

One hypothesis to explain our results is related to the fact that the CNS has a neuronal excess at the birth, that under normal conditions is progressively lost in parallel to the process of myelination and synaptic specialization [33, 34]. It means that ontogenetic stages occurred during the gestation (cell proliferation, neural migration, selective aggregation, cellular differentiation, and synaptogenesis) resulted from the excess of neurons, neuronal circuits, and synapses. Therefore, the normal development of the nervous system can also include the subtractive or regression events, that is, axonal retraction, synaptic degeneration, and neuronal death. This neuronal death (apoptosis) is genetically programmed and has exerted physiological functions [33, 34].

Another important hypothesis refers to the occurrence of a distortion, as the paradigm employed (severe pre-gestational diabetes) led the increase of fetal death, so the remaining offspring can be the more benefited by the related neuroplastic phenomena.

## 5. Conclusions

The results presented herein did not allow us to affirm that severe pregestational diabetes does not impair the neurofunctional status of male offspring, pointing to the need of further investigation.

##  Conflict of Interests 

The authors declare no conflict of interests.
